# Central Autonomic Network Regions and Hypertension: Unveiling Sympathetic Activation and Genetic Therapeutic Perspectives

**DOI:** 10.3390/biology12081153

**Published:** 2023-08-21

**Authors:** Vera Geraldes, Sérgio Laranjo, Catarina Nunes, Isabel Rocha

**Affiliations:** 1 Cardiovascular Centre of the University of Lisbon, 1649-028 Lisbon, Portugal; vgeraldes@medicina.ulisboa.pt (V.G.); catraq@live.com.pt (C.N.); 2 Institute of Physiology, Faculty of Medicine of the University of Lisbon, 1649-028 Lisbon, Portugal; 3 NOVA Medical School, Universidade NOVA de Lisboa, 1169-056 Lisboa, Portugal

**Keywords:** rostral ventrolateral medulla, lateral parabrachial nucleus, periaqueductal grey matter, essential hypertension, sympathoexcitation, baroreceptor reflex, chemoreceptor reflex, lentivirus, genetic therapy

## Abstract

**Simple Summary:**

High blood pressure, or hypertension, is a serious condition with potentially life-threatening consequences. Researchers conducted a study to explore how certain areas of the brain contribute to high blood pressure. The focus was on three specific brain regions: the lateral parabrachial nucleus (LPBN), Kolliker-fuse nucleus (KF), and periductal grey matter (PAG), which play roles in regulating blood pressure. Using genetic modification techniques, the researchers decreased the neuronal activity in these brain regions. They observed interesting outcomes: reducing activity in the LPBN led to significant decreases in blood pressure and heart rate; in the KF, the activity of the nerves involved in blood pressure regulation and breathing decreased; however, reducing activity in the PAG did not have a significant impact on blood pressure, but affected the heart’s response to changes in blood pressure. These findings highlight the importance of specific brain areas, particularly the LPBN, in regulating blood pressure. While this study was conducted on rats and further research is needed to apply these findings to humans, it provides valuable insights into the complex factors contributing to high blood pressure. This knowledge could potentially pave the way for future treatments and interventions targeting these brain regions in order to better manage hypertension.

**Abstract:**

Introduction: Hypertension, a leading cause of death, was investigated in this study to understand the role of specific brain regions in regulating blood pressure. The lateral parabrachial nucleus (LPBN), Kolliker-fuse nucleus (KF), and periductal grey matter (PAG) were examined for their involvement in hypertension. Methods: Lentiviral vectors were used to alter the activity of these brain regions in hypertensive rats. Over a 75-day period, blood pressure, heart rate, reflex responses, and heart rate variability were measured. Results: Decreasing the activity in the LPBN resulted in a reduced sympathetic outflow, lowering the blood pressure and heart rate. In the KF, the sympathetic activity decreased and chemoreflex variation was attenuated, without affecting the blood pressure. Silencing the PAG had no significant impact on blood pressure or sympathetic tone, but decreased cardiac baroreflex gain. Discussion: These findings highlight the significant role of the LPBN in hypertension-related sympathetic activation. Additionally, LPBN and KF neurons appear to activate mechanisms that control respiration and sympathetic outflow during chemoreceptor activation. Conclusions: The study provided insights into the contribution of the midbrain and pontine regions to neurogenic hypertension and offers potential avenues for future genetic interventions and developing novel treatment approaches.

## 1. Introduction

Hypertension, often referred to as the silent disease, is a significant cause of premature death, affecting approximately 1.13 billion people worldwide. This global health concern is prevalent in one in four men and one in five women [1]. Most of these cases are attributed to essential hypertension of neurogenic origin. Hyperactivity of the sympathetic nervous system plays a crucial role during the onset, development, and maintenance of essential hypertension, a phenomenon that is well established in Dahl salt-sensitive deoxycorticosterone-saline and spontaneously hypertensive rat (SHR) models of hypertension [1,2,3].

The paraventricular nucleus of the hypothalamus (PVN) and rostroventrolateral medulla (RVLM), known as the sympathoexcitatory areas, are involved in blood pressure control. These areas contain a significant number of neurones that are activated under hypertensive conditions [4,5,6,7]. These sympathoexcitatory areas send efferent fibres to other central autonomic nuclei, such as the lateral parabrachial nucleus (LPBN), Kolliker-fuse (KF) nucleus, and periductal grey matter (PAG), which participate in cardiovascular and respiratory control [8,9,10,11,12].

The LPBN contributes to increased sympathetic nervous system function associated with hypertension. Increases or decreases in the arterial blood pressure promote elevated levels of Fos-immunoreactive neurones in the central and dorsal subnuclei of the LPBN [8,13,14,15]. In contrast, a temporary chemical inactivation of the LPBN induces a pressor response [16], and electrolytic lesions enhance baroreflex-mediated cardiovascular responses while their activation is inhibited [17,18]. Another study showed that ablation of the LPBN produces a significant transient reversal of renal-wrap hypertension [19,20]. 

In addition to the LPBN, the KF nucleus, which is primarily involved in central respiratory control, also participates in cardiovascular control [21,22,23,24]. Electrical stimulation of the KF causes a pressor effect with mild tachycardia, and a KF blockade changes the cardiovascular responses produced by glutamate injections into the cuneiform nucleus [25]. However, another study concluded that synapses in the KF nucleus do not affect the regulation of the basal blood pressure and heart rate [26].

The PAG, another autonomic area, also plays a role in cardiovascular control. Activation of the PAG elicits both respiratory and cardiovascular responses [24,27,28]. Dorsal PAG electrical stimulation produces a pressor response in rats with tachycardia, vasodilatation in the hindlimb, and hyperpnea, similar to a defence reaction response [29,30]. Another study showed that electrolytic lesions of the PAG promote a significant decrease in cardiac baroreflex gain and resting mean blood pressure, and a moderate increase in heart rate 24 h after lesions in adult SHRs [31].

Despite the known roles of these autonomic areas in hypertension, the precise mechanisms by which they contribute to the disease remain unclear. To address this gap, we used a virus expressing a potassium channel to depress the electrical excitability of these neurones. In previous studies, we showed that lentiviral vector (LVV) overexpression of inward-rectifying potassium channels (hKir2.1) depressed the electrical excitability of the PVN and RVLM neurones [6,7,32]. In fact, decreasing the PVN and RVLM neuronal excitability, induced by the overexpression of hKir2.1, reduced the blood pressure and sympathetic output, reversed cardiorespiratory reflexes in the PVN, and improved the baroreflex function in the RVLM of SHRs [6,7].

In this study, we aimed to elucidate the role of the LPBN, KF nucleus, and PAG in the genesis and maintenance of hypertension, using a novel approach of genetic modulation with lentivirus. Our findings provide new insights into the mechanisms of essential hypertension and may potentially inform the development of novel therapeutic strategies. We hypothesised that the overexpression of hKir2.1 in these autonomic areas would depress neuronal excitability, leading to a decrease in the sympathetic outflow and blood pressure in SHRs, thereby providing a new perspective on the neurogenic origin of essential hypertension.

## 2. Materials and Methods

### 2.1. Ethical Considerations

All the experimental procedures in this study adhered to the European Directive 2010/63/EU and Portuguese Law 113/2013 on animal welfare. The study protocol was approved by the Ethics Committee of the Lisbon Medical School, University of Lisbon, Portugal. 

### 2.2. Animals

This study was conducted on both male and female spontaneously hypertensive rats (SHRs, *n* = 30) aged > 10 weeks. The rats were obtained from Charles River (Spain). They were housed individually at the Faculty of Medicine animal house in a temperature- and humidity-controlled room (20 °C and 55%, respectively) and synchronised to a 12 h–12 h light/dark cycle. The rats were provided with standard rat chow ad libitum and had free access to tap water.

### 2.3. Metabolic Evaluation

Before the commencement of the microinjection protocol and 59 days later, the animals were individually housed for 24 h in metabolic cages. The rats were given free access to 200 g of standard rat chow and 250 mL of water. The evaluated parameters included food and fluid intake, urine and faecal production, and body weight. Water waste was also considered. Food and fluid intake was measured by weighing the food and water before and after the 24 h period, as well as the produced urine and faeces after the 24 h period.

### 2.4. Surgical procedures

#### 2.4.1. Implantation of Radio-Telemetry Probes

Following the administration of anaesthesia (ketamine, 100 mg.mL^−1^, and dexmedetomidine, 0.5 mg.mL^−1^, IP), a medial laparotomy was performed. The abdominal aorta was clamped proximally and a radio-telemetry sensor catheter (approximately 0.7 mm, thin-walled thermoplastic membrane) was inserted into the root of the abdominal aorta, between the renal and iliac arteries, under the guidance of a binocular microscope. The catheter and artery were secured with a cellulose patch and tissue adhesive. A radio-telemetric pressure transducer (DSI, St. Paul, MN, USA), a fluid-filled catheter connected to a PA-C40 transmitter, was sutured to the abdominal wall. The internal and skin layers were closed with polyglycolic acid and silk surgical sutures, respectively. At the end of the surgical procedure, an anti-inflammatory (carprofen, 4 mg.kg^−1^, SC) and sedative/analgesic reverser (atipamezole, 5 mg mL^−1^, IM) were administered. A recovery period of at least 15 d was allowed before the experiment.

#### 2.4.2. Viral Vector Construction and Validation

The construction of the lentiviral vector was based on previous studies [19,20,21]. Briefly, LVV-hKir2.1 is a mix of LV-TREtight-Kir-cIRES-GFP 5.4 × 10^9^ IU and LV-Syn-Eff-G4BS-Syn-Tetoff 6.2 × 10^9^ IU (ratio 1:4), which expresses eGFP, as well as human inwardly rectifying potassium channels of the hKir2.1 type in neuronal cells. LVVeGFP, used for the SHAM group (used as a control group to prevent bias, involving a procedure that mimicked the experimental intervention without delivering the active treatment), was a mixture of LVTREtight-GFP (5.7 × 10^9^ IU) and LV-Syn-Eff-G4BS-Syn-Tetoff (6.2 × 10^9^ IU) in a 1:4 ratio. This binary system expresses eGFP. The validation of the transduction and transgene expression efficacy was performed as described previously and included mRNA expression, immunocytochemical, and electrophysiological data [19,20,21].

#### 2.4.3. Central Microinjection Sites

After the implantation of the catheter, the animals were randomly assigned to one of three groups according to the microinjected area: the Lateral Parabrachial Nucleus (LPBN) (n = 10), Kolliker-Fuse nucleus (KF) (n = 10), or Periaqueductal Grey Matter (PAG) (n = 10). All of these central brain regions were compared to control animals used in previous studies in our laboratory, thus applying 3R rules, where we demonstrated that LVV-eGFP did not elicit alterations in the cardiovascular variables and sympathetic output [6,7]. Two weeks after the radio-telemetry probe implantation, the rats were re-anaesthetised (ketamine, 100 mg.mL^−1^, IP; dexmedetomidine, 0 mg/mL, i.p.) and placed on a stereotactic frame (Kopf Instruments, Tujunga, CA, USA). Upon a craniotomy, a bilateral microinjection of LVV-hKir2.1 (0.05 μL) into the aforementioned brain regions was performed (LPBN—Bregma: −9.8 mm, Lateral: 2.4 mm, and Deep: 6.8 mm) ( KF—Bregma: −8.7 mm, Lateral: 2.6 mm, and Deep: 7.8 mm) (PAG—Bregma: −5.2 mm, Lateral: 0.5 mm, and Deep: 5.4 mm) [22]. The volume of the microinjection was carefully controlled to limit the transduction to the confines of the chosen areas. The rats were allowed to recover and monitored via telemetry for 60 days.

#### 2.4.4. Cardiorespiratory Evaluation

On the 60th day, the animals were re-anaesthetised (sodium pentobarbitone, 60 mg.kg^−1^, IP), and the trachea, common carotid artery, femoral artery, and vein were cannulated. A heating blanket was used to monitor the rectal temperature (Harvard Apparatus). The ECG was recorded, and the heart rate (HR) was derived from this. The baroreceptor reflex was activated via phenylephrine administration (0.2 mL, 25 μg.mL^−1^ iv; Sigma Aldrich, St. Louis, MO, USA). Retrogradely injected lobeline (0.2 mL, 25 μg.mL^−1^ iv, Sigma Aldrich, St. Louis, MO, USA) through the external carotid artery triggered the peripheral chemoreceptor reflex. The baro- and peripheral chemoreceptor reflexes were activated twice, with a 5 min interval between the stimulations. The ECG, HR, and BP (systolic, diastolic, and mean) were continuously recorded throughout the experiment.

### 2.5. Morphological Studies

After the cardiorespiratory reflex evaluation, the animals were terminally anaesthetised with an overdose of anaesthetic (sodium pentobarbitone, 60 mg.kg^−1^, i.v.), and the brain areas of interest for each group were rapidly removed, placed on Tissue-Tek^®^, and frozen with isopentane and liquid nitrogen at −80 °C. The coronal sections (18 µm) were cut using a freezing microtome and mounted on slides. Using an epifluorescence microscope, the eGFP-labelled regions were identified and plotted on standardised sections of the Paxinos and Watson atlas [22].

### 2.6. Data Acquisition and Analysis

With a 10-day interval for 60 days, telemetric data were acquired at 1 kHz and analysed using suitable software (LabChart6, Powerlab, ADInstruments, Colorado Springs, CO, USA). The blood pressure, heart rate, and respiratory rate were also measured.

#### 2.6.1. Baroreceptor, Chemoreceptor Reflex, and Overall Autonomic Tone Evaluation

1. *Baroreceptor Reflex Evaluation:* The baroreceptor reflex gain (BRG) was quantified using the following formula: BRG = (HRbasal-HRBPmax)/(BPmax-BPbasal) (bpm mmHg ^−1^). This calculation provides a measure of the baroreceptor reflex sensitivity, which is a key mechanism for short-term blood pressure regulation [23].
2. *Chemoreceptor Reflex Evaluation*: The chemoreceptor reflex (ChR) was evaluated by measuring the change in the respiratory rate (∆ChR) in response to lobeline stimulation. The respiratory rate (RespR) was derived from the tracheal pressure before and after the lobeline administration. The following formula was used: ∆ChR = RespRlobeline-RespRbasal [24].
3. *Autonomic Tone Evaluation*: To assess the overall autonomic tone, a spectral analysis of the systolic blood pressure (SBP) and RR interval data was performed. The low-frequency (LF) band (0.15–0.6 Hz) of the SBP was used as an indicator of the sympathetic activity, while the high-frequency (HF) band (0.6–2.0 Hz) of the RR interval was used as an indicator of the parasympathetic activity. The data were analysed in the frequency domain using a Fast Fourier Transform with the in-house software Fisiosinal [25].
4. *Respiratory Sinus Arrhythmia*: Respiratory sinus arrhythmia, a measure of parasympathetic activity, was quantified as the ratio of the longer RR interval of the ECG during expiration to the shorter RR interval during inspiration, as described previously [26].

#### 2.6.2. Circadian BP and HR Profile

The circadian patterns of the blood pressure (BP) and heart rate (HR) were analysed by segregating the telemetric data recordings into light (7 AM–7 PM) and dark (7 PM–7 AM) phases. The mean BP and HR values were calculated for each phase. The light–dark transition, which is a significant aspect of the circadian rhythm in rodents, was considered. This analysis was designed to identify any significant alterations in the circadian rhythms of the BP and HR following the microinjection. Differences in the mean values of the BP and HR between the light and dark phases were statistically analysed to determine the impact of the intervention on the circadian patterns of these parameters.

### 2.7. Statistical Analysis

Statistical analyses were conducted using the latest version of the Statistical Package for the Social Sciences (SPSS). Within-group and between-group comparisons were performed. For the within-group comparisons, specifically before and after the microinjection, a paired Student’s *t*-test was used. A one-way analysis of variance (ANOVA) was employed for the between-group comparisons during the same period. Prior to these analyses, the normality of the data distribution was verified using the Shapiro–Wilk test. All the data are presented as the mean ± standard error of the mean (SEM). Statistical significance was set at *p* < 0.05.

## 3. Results

### 3.1. Lateral Parabrachial Nucleus

#### 3.1.1. LPBN Lentiviral Microinjection Influence on Long-Term Blood Pressure Control

**Figure 1:**
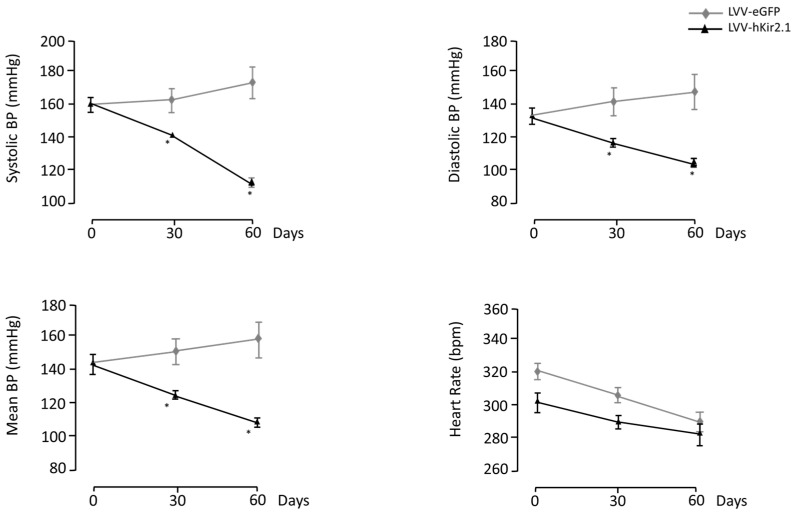
The lentiviral microinjection of LVV-hKir2.1 into the LPBN evoked a significant decrease in systolic, diastolic, and mean blood pressure and heart rate of SHR rats (n = 10; * *p* < 0.05). Control animal parameters followed the expected tendency to increased values due to the physiological characteristics of the animal model development.

Before the LVV-hKir2.1 bilateral microinjection into the LPBN (n = 8), the basal blood pressure (BP) values were 161 ± 1, 133 ± 1, and 142 ± 1 mmHg for the sBP, dBP, and mBP, respectively. The basal BP values for the SHR LVV-eGFP group (n = 10) were 160 ± 2 mmHg for the sBP, 135 ± 1 mmHg for the dBP, and 143 ± 2 mmHg for the mBP. All the BP values decreased significantly at 60 days after the microinjection into the LPBN compared to the LVV-eGFP SHRs (111 ± 1, 104 ± 1, and 107 ± 1 mmHg vs. 174 ± 10, 149 ± 11, and 157 ± 10 mmHg, respectively, n = 10, *p* < 0.05) (Figure 1). The heart rate values also decreased 60 days after the microinjection (323 ± 1 vs. 291 ± 3 bpm, *p* < 0.0001) (Figure 1). 

**Figure 2:**
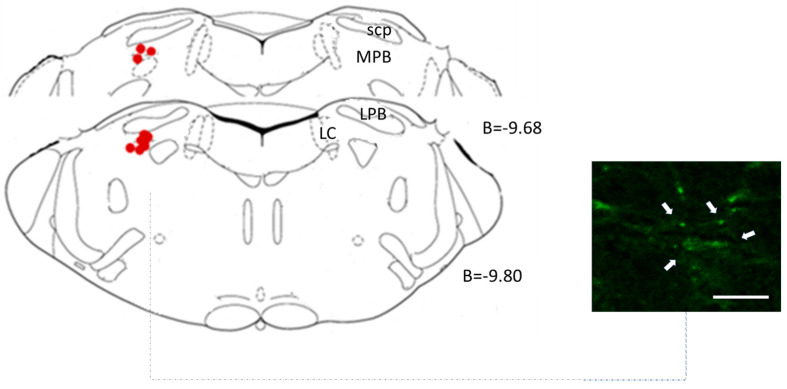
Illustration of targeted regions within the LPBN for lentiviral microinjection (red dots). The delineated areas indicate the specific points of lentiviral vector insertion, offering a comprehensive spatial perspective of the experiment. B-bregma; LC-Locus coeruleus; LPB-lateral parabrachial nucleus; scp-superior cerebellar peduncle (plates based on Paxinos and Watson [22]). The scale bar (white arrow) measures 50 μm.

A representative section of the lentiviral microinjection into LPBN is shown in Figure 2.

#### 3.1.2. LPBN Lentiviral Microinjection Impact on Sympathetic Tone

Fast Fourier Transform application to the sBP and interpulse intervals revealed that the cardiovascular autonomic outflow of the SHR LVV-hKir2.1 animals significantly decreased 60 days post-microinjection, both within the same group amongst day 0 and day 60 (SHR LVV-hKir2.1: day 0–0.11 ± 0.002 mmHg^2^ms^−2^ and day 60–0.05 ± 0.004 mmHg^2^ms^−2^, *p* < 0.05, n = 10), and between groups (SHR LVV-eGFP: day 0–0.08 ± 0.02 mmHg^2^ms^−2^ and day 60–0.08 ± 0.003 mmHg^2^ms^−2^, *p* < 0.05, n = 10). This autonomic outflow decrease was due to a fall in the sympathetic output expressed by the LF band power (SHR LVV-hKir2.1: day 0–1.61 ± 0.07 mmHg^2^ms^−2^ vs. day 60–0.73 ± 0.04 mmHg^2^ms^−2^; *p* < 0.05). The SHR LVV-eGFP animals did not differ significantly within the group, but did so with the LVV-hKir2.1 one (SHR LVV-eGFP: day 0–0.86 ± 0.12 mmHg^2^ms^−2^ vs. day 60–0.86 ± 0.19 mmHg^2^ms^−2^; *p* < 0.05 compared to LVV-hKir2.1 rats).

#### 3.1.3. Blood Pressure and Heart Rate Circadian Variation 

Under basal conditions, the BP circadian variation profile followed a similar trend, with lower BP values observed during the light phase than those during the dark phase. As expected, the HR was lower during the light phase.

On the 60th day, the LPBN LVV-hKir2.1 animal group showed a non-significant increase in sBP, dBP, and mBP, accompanied by a decrease in HR during both the light and dark phases (light phase_day 0_sBP—160 ± 5 mmHg, dBP—131 ± 6 mmHg, mBP—141 ± 6 mmHg, and HR—299 ± 13 bpm vs. day 60_ sBP—166 ± 6 mmHg, dBP—131 ± 13 mmHg, mBP—143 ± 7 mmHg, and HR—277 ± 9 bpm; dark phase_day 0_sBP—158 ± 1 mmHg, dBP—127 ± 2 mmHg, mBP—138 ± 1 mmHg, and HR—325 ± 9 bpm vs. day 60_ sBP—176 ± 6 mmHg, dBP—141 ± 13 mmHg, mBP—153 ± 7 mmHg, and HR—329 ± 15 bpm, *p* > 0.05). The systolic, diastolic, and mean BP plus HR light-phase values for the SHR LVV-eGFP group were 158 ± 4 mmHg, 133 ± 5 mmHg, 148 ± 5 mmHg, and 292 ± 6 bpm vs. 171 ± 11 mmHg, 145 ± 10 mmHg, 154 ± 10 mmHg, and 264 ± 5 bpm, for days 0 and 60, respectively. The dark-phase values followed a similar trend (*p* > 0.05).

#### 3.1.4. Parasympathetic Tonus Indirect Assessment

Respiratory sinus arrhythmia (RSA) was used as an index for the vagal control of the heart. In our research project, the LVV-hKir2.1 microinjection into the LPBN did not affect the RSA. The LVV-hKir2.1 rats presented similar RSA profiles on days 0 and 60 (1.07 ± 0.76 vs. 1.03 ± 0.73, *p* > 0.05). Likewise, the RSA values for the SHR LVV-eGFP group varied from 1.06 ± 0.01 to 1.03 ± 0.01 (*p* > 0.05), suggesting that the vagal tonus was unaffected by the LPBN’s decreased excitability.

#### 3.1.5. Cardiovascular Reflexes Evaluation

**Figure 3:**
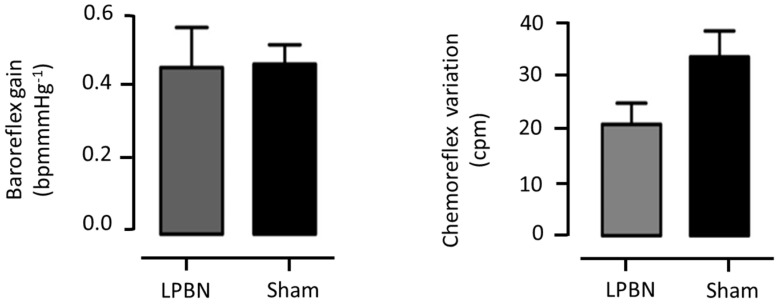
LVV-hKir2.1 microinjection into LPBN effect on baroreflex gain and chemoreflex variation, 60 days after microinjection.

No significant changes were observed in the baroreflex gain between both groups (0.45 ± 0.1 vs. 0.46 ± 0.05 bpm/mmHg; LPBN and sham, correspondingly, *p* > 0.05) (Figure 3).

Regarding the peripheral chemoreceptor reflex, and despite the different variations between them, full statistical significance was not attained (LPBN: Δ19.7 ± 3.7 vs. Sham: Δ31.5 ± 4.5 cpm; *p* > 0.05, n = 10) (Figure 3). 

### 3.2. Periaqueductal Gray Matter

#### 3.2.1. PAG Lentiviral Microinjection Influence on 24 h Mean Values of Blood Pressure and Heart Rate

**Figure 4:**
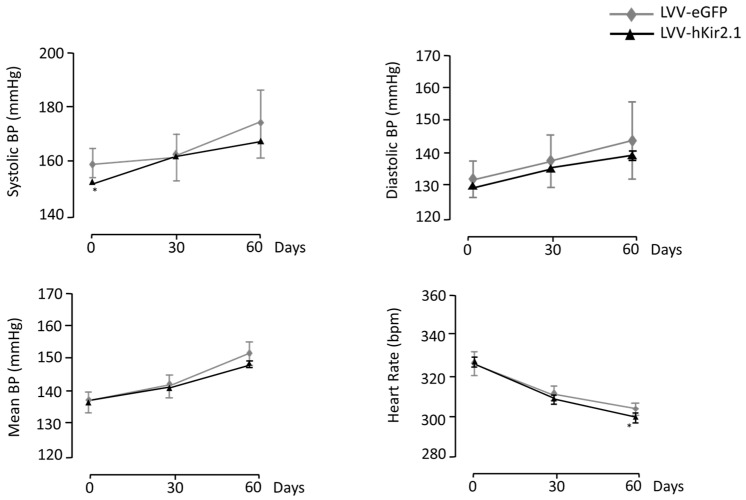
Effect of lentiviral microinjection of LVV-hKir2.1 into the PAG on systolic, diastolic, and mean blood pressure and heart rate, for a 60 day period. * *p* < 0.05, comparison between day 0 and day 60 of LVV-hKir2.1 animals.

The basal blood pressure values before the LVV-hKir2.1 bilateral microinjection into the PAG (n = 10) were 152 ± 1 for the sBP, 130 ± 1 for the dBP, and 137 ± 1 mmHg for the mBP. There were no significant differences between the sham and PAG LVV-hKir2.1 animals. Sixty days after the lentiviral microinjection, the PAG LVV-hKir2.1 sBP, dBP, and mBP were 168 ± 1, 144 ± 5, and 152 ± 4 mmHg, respectively, demonstrating that the lentiviral microinjection did not alter the BP values (n = 10; *p* > 0.05). Statistical significance was obtained to compare the sBP between days 0 and 60, which increased during the protocol period. The heart rate decreased significantly (*p* < 0.05), thus accompanying an increase in the BP (Figure 4). 

**Figure 5:**
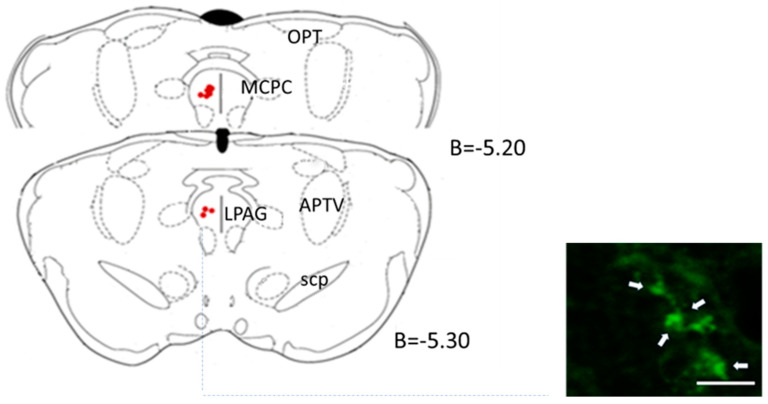
Representation of PAG region targeted for lentiviral microinjection (red dots). The marked regions represent the precise locations where the lentiviral vector was delivered, B-bregma; APTV-anterior pretectal nucleus, ventral part; LPAG-lateral periaqueductal grey; MCPC-magnocellular nucleus of the posterior commissure; OPT-olivary pretectal nucleus; Scp-superior cerebellar peduncle; (plates adapted from Paxinos and Watson [22]). Scale bar (white arrow) represents 25 μm.

Despite not directly affecting the blood pressure or heart rate, Figure 5 displays a representative section highlighting the site of the lentiviral microinjection.

#### 3.2.2. PAG Lentiviral Microinjection Effect on Sympathetic Output

The sympathetic tone was assessed indirectly by applying a fast Fourier Transform to the sBP and RR intervals. The PAG LVV-hKir2.1 animals exhibited no change in their LF(BP)/HF(RR) ratio, i.e., the cardiovascular autonomic outflow was not altered 60 days post-LVV-hKir2.1-microinjection (0.04 ± 0.07 vs. 0.05 ± 0.02 mmHg^2^ms^−2^, *p* > 0.05) when compared to the sham rats (0.08 ± 0.02 vs. 0.09 ± 0.03 mmHg^2^ms^−2^, *p* > 0.05). 

#### 3.2.3. Blood Pressure and Heart Rate Circadian Variation

On the 60th day, the PAG LVV-hKir2.1 animal group showed a significant increase in their systolic, diastolic, and mean BPs during both the light and dark phases (Table 1, n = 10, *p* < 0.05). A decrease in the HR was observed in both stages. The SHR LVV-eGFP group’s sBP, dBP, and mBP values for the light and dark phases at 60 days were similar to those of the PAG LVV-hKir2.1 group (Table 1, n = 10, *p* > 0.05).

#### 3.2.4. Indirect Quantification of Vagal Tonus

Respiratory sinus arrhythmia, an index of the vagal tonus, was not changed by the LVV-hKir2.1 microinjection in the PAG (1.07 ± 0.01 vs. 1.24 ± 0.1, *p* > 0.05). In addition, the SHR LVV-eGFP group’s vagal tonus remained the same (1.05 ± 0.01 to 1.05 ± 0.01 *p* > 0.05). Overall, the vagal tonus was not affected by a decrease in the excitability of the PAG.

#### 3.2.5. Cardiorespiratory Evaluation

**Figure 6:**
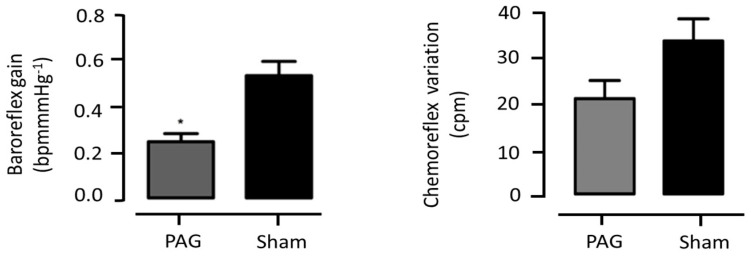
LVV-hKir2.1 microinjection into PAG effect on baroreflex gain and chemoreflex variation, 60 days after microinjection. Lentiviral administration further impaired baroreflex sensitivity. * *p* < 0.05, statistically significant difference between PAG and SHAM groups.

A significant fall in the baroreflex gain (BRG) of the PAG LVV-hKir2.1 group was observed when compared to that of the sham group (0.24 ± 0.03 vs. 0.51 ± 0.05 bpm/mmHg; *p* < 0.05, n = 10) (Figure 4). Contrarily, the chemoreflex variation did not differ significantly between both groups (∆22 ± 5.6 vs. ∆32.6 ± 5.1 cpm; *p* > 0.05, n = 10) (Figure 6).

### 3.3. Kolliker-Fuse Nucleus

#### 3.3.1. KF Lentiviral Microinjection Influence on 24 h Mean Values of Blood Pressure and Heart Rate

**Figure 7:**
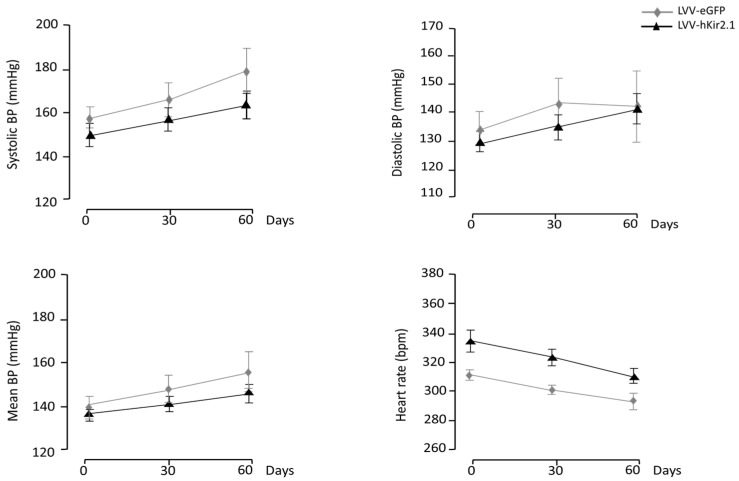
Effect of lentiviral microinjection of LVV-hKir2.1 into the KF on systolic, diastolic, and mean blood pressure and heart rate, for a 60 day period. LVV-hKir2.1 administration did not reflect itself in blood pressure values. No significant changes were found.

The KF LVV-hKir2.1 group’s basal blood pressure values were 150 ± 4 mmHg for the sBP, 129 ± 4 mmHg for the dBP, and 136 ± 3 mmHg for the mBP. No significant differences were found between the KF and sham groups (158 ± 5, 133 ± 6, and 141 ± 6 mmHg, respectively; *p* > 0.05, n = 10) at the beginning of the protocol (Figure 7).

**Figure 8:**
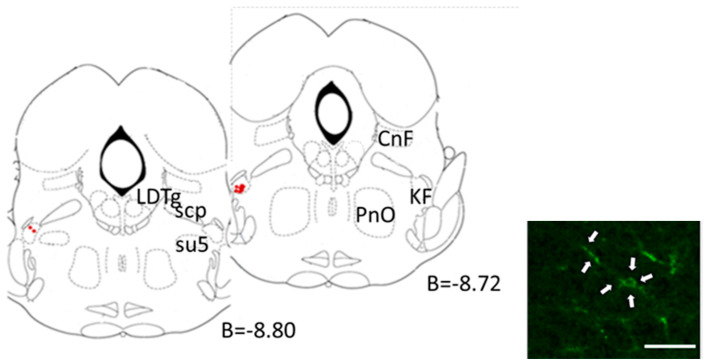
Representation of target KF areas for lentiviral microinjection (red dots). The highlighted zones depict the exact sites of lentiviral vector administration, providing a detailed spatial overview of the experimental process. B-bregma; CnF-cuneiform nucleus; KF-Kolliker Fuse nucleus; LDTg-laterodorsal tegmental nucleus; PnO-pontine reticular nucleus; scp-superior cerebellar peduncle; Su5-supratrigeminal nucleus (plates adapted from Paxinos and Watson [22]). The scale bar (white arrow) indicates 50 μm.

Sixty days after the lentiviral microinjection, the KF LVV-hKir2.1 sBP, dBP, and mBP were 164 ± 6, 140 ± 5, and 148 ± 5 mmHg, respectively, indicating that the LVV-hKir2.1 microinjection did not elicit any change in the blood pressure values of the SHR KF group (*p* > 0.05). The heart rate decreased 60 days post-microinjection (322 ± 16 vs. 301 ± 7 bpm, *p* > 0.05) (Figure 7). Figure 8 illustrates a representative cross-section where a lentiviral microinjection was administered.

#### 3.3.2. Effect of KF LVV-hKir2.1 Microinjection on Sympathetic Output

The rats microinjected with LVV-hKir2.1 into the KF showed a notable decrease in the sympathetic output expressed by the LF band power and measured indirectly through Fast Fourier Transform (KF LVV-hKir2.1 day 0:0.84 ± 0.34 mmHg^2^ms^−2^ vs. day 60:0.46 ± 0.18 mmHg^2^ms^−2^ and SHR LVV-eGFP day 0:0.86 ± 0.14 mmHg^2^ms^−2^ vs. day 60:0.71 ± 0.16 mmHg^2^ms^−2^, n = 10, *p* < 0.05 for comparisons between groups). Owing to the previous, the cardiovascular autonomic outflow also decreased 60 days post-microinjection when the values were compared within the same group (KF LVV-hKir2.1 day 0:0.16 ± 0.1 mmHg^2^ms^−2^ and day 60:0.02 ± 0.01 mmHg^2^ms^−2^, n = 10, *p* < 0.05). Moreover, a comparison between day 60 of the KF LVV-hKir2.1 and SHR LVV-eGFP groups revealed a significant decrease between the groups (SHR LVV-eGFP day 0:0.06 ± 0.02 mmHg^2^ms^−2^ and day 60:0.06 ± 0.01 mmHg^2^ms^−2^, n = 10, *p* < 0.05).

#### 3.3.3. Blood Pressure and Heart Rate Circadian Variation

As anticipated, lower BP values were observed during the light phase than the dark phase. The HR was higher during the dark stage. Sixty days after the microinjection into the KF, LVV-hKir2.1 did not affect the systolic, diastolic, or mean BP, indicating that all the BP values increased during both the light and dark phases (light-phase LVV-hKir2.1 d 0: sBP_148 ± 2, dBP_127 ± 3, and mBP_134 ± 3 mmHg vs. LVV-hKir2.1 d 60: sBP_161 ± 6, dBP_138 ± 5, and mBP_146 ± 5 mmHg; dark-phase LVV-hKir2.1 d 0: sBP_152 ± 5, dBP_131 ± 4, and mBP_138 ± 4 mmHg vs. LVV-hKir2.1 d 60: sBP_166 ± 7, dBP_142 ± 6, and mBP_150 ± 6 mmHg; n = 8, *p* > 0.05) (Table 2). The SHR sham values for the systolic, diastolic, and mean BP showed similar behaviour to those of the KF, LVV-hKir2.1 (n = 10, *p* > 0.05) (Table 2). 

#### 3.3.4. Indirect Assessment of Vagal Tonus

An indirect assessment of the parasympathetic tonus via a respiratory sinus arrhythmia quantification revealed that the lentiviral microinjection into the KF did not elicit any modification in the vagal tonus of the animals (1.14 ± 0.01 vs. 1.18 ± 0.5, *p* > 0.05). The SHR LVV-eGFP rats’ parasympathetic outflow was also unchanged (1.03 ± 0.01 to 1.04 ± 0.01 *p* > 0.05).

#### 3.3.5. Cardiorespiratory Reflex Assessment

**Figure 9:**
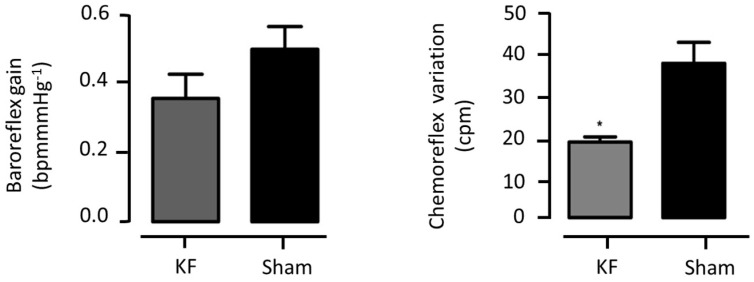
LVV-hKir2.1 microinjection into KF effect on baroreflex gain and chemoreflex variation, 60 days after microinjection. Lentiviral administration generated a decrease in chemoreflex variation of KF animals, compared to sham ones. * *p* < 0.01, value with significant statistical difference between experimental groups.

The KF and sham group baroreflex gain did not differ significantly between the groups (0.37 ± 0.07 vs. 0.47 ± 0.04 bpm/mmHg; *p* > 0.05), although there seemed to be a tendency towards a BRS reduction in the KF LVV-hKir2.1 group (Figure 9).

On the other hand, the KF animals exhibited a significant decrease in their chemoreflex variation when compared to that of the sham ones (Δ17.5 ± 1.3 vs. Δ36.2 ± 4.8 cpm; n = 10, *p* < 0.01). This was expected due to the functional nature of the KF in the pontine control of respiration (Figure 9). 

## 4. Discussion

The role of the brain, particularly the central autonomic network, has been increasingly recognised in the complex landscape of essential hypertension. The intricate interplay between the various brain regions involved in autonomic regulation contributes to the development and maintenance of hypertension. However, the exact mechanisms and interactions involved in this remain unclear. In this study, we aimed to elucidate these mechanisms by investigating the roles of specific brain autonomic areas related to cardiorespiratory regulation. We focused on the midbrain and pontine regions, which are implicated in the control of the sympathetic activity and blood pressure. By genetically modulating the cell excitability to decrease the activity of these areas, we assessed the long-term effects on the blood pressure, peripheral sympathetic tone, and cardiovascular reflex responses in an animal model of essential hypertension. The following discussion elaborates our findings and their implications for understanding the pathophysiology of essential hypertension.

The LPBN, KF nucleus, and PAG are brain regions that form interconnected neural circuits involved in the regulation of the cardiovascular and respiratory functions. They receive inputs from various brain centres and sensory information from the periphery, such as baroreceptor and chemoreceptor inputs, integrating this information to modulate the autonomic outflow and respiratory patterns.

The LPBN and PAG silencing revealed different autonomic mechanisms, indicating that these areas regulate the sympathetic activity and blood pressure differently. The established roles for these two areas indicate that the PAG is more involved in the relationship between nociception and autonomic behaviour than the LPBN, which is a primary pontine relay for visceral autonomic information with strong neuronal links to the nucleus tractus solitarius, hypothalamus, and RVLM. 

Interestingly, the baroreflex gain decreased under the PAG silencing, mainly because of a decrease in the HR, which was significantly achieved. Visceral reflexes influence nociceptive processing, and pain-evoked potentials decrease under hypertensive conditions and upon baroreceptor stimulation [27]. However, pain evaluation was not the purpose of this study, but the role of the PAG in sympathoexcitation. Some studies on normotensive animals have shown that PAG stimulation produces a cardiovascular response characterised by sympathetic overactivity [28,29,30]. Cardiac baroreflex gain is markedly attenuated after electrolytic lesions in the dorsolateral PAG [30]. This suggests that the PAG plays a crucial role in baroreflex mediation, and its silencing disrupts the blood pressure cardiovascular reflex control. 

PAG columns can modulate the cardiac sympathetic function through indirect pathways involving sympathetic premotor neurones found at specific sites in the hypothalamus, midbrain, pons, and medulla oblongata, modulating various physiological functions, including cardiovascular and respiratory activities. Additionally, the major outflow of the PAG terminates in the bulbospinal regions of the RVLM. Therefore, PAG silencing results in a decrease in the sympathetic activity, which activates adjacent sympathetic areas via its projections [28,29,30]. Schenberg et al. showed that bilateral electrolytic lesions of the PAG evoke baroreflex gain attenuation [31]. The latter is evidence of the PAG’s significant influence on the resting cardiovascular control in SHRs. In our study, PAG silencing also elicited a diminished cardiac baroreflex gain, and we hypothesised that this was due to its effects on the RVLM, where decreased sympathetic activity in the first resulted in little stimulation of the latter, and thus less blood pressure control.

Despite the decreased baroreflex gain, the LVV-hKir2.1 PAG microinjection did not affect the peripheral chemoreflex-evoked cardiovascular and respiratory responses. Concerning the neuronal pathways of the chemoreflex, there is evidence that other areas in the pons and midbrain receiving projections from the NTS may be involved in the autonomic and humoral responses to chemoreflex activation [28,32]. In fact, a depressor response was produced during an optogenetic stimulation of the NTS PNMT neurons projecting to the paraventricular nucleus of the hypothalamus, lateral parabrachial nucleus, and caudal ventrolateral medulla [33]. Recently, we verified that decreasing the excitability of the paraventricular nucleus of the hypothalamus (PVN) produced a marked reduction in the chemoreflex sensitivity, indicating that this nucleus may play a permissive role in the sympathoexcitatory component of the chemoreflex [7].

Decreasing the excitability of the LPBN evoked a decline in the sympathetic activity, a decrease in the BP without HR changes, changes in the circadian BP, and in the carotid chemoreflex but not the baroreceptor reflex. Cardiac baroreflex gain, impaired in the SHRs compared to the normotensive rats, depends on the HR and BP. Since there were no changes in the HR after the lentiviral microinjection, this conditioned the expected baroreflex improvement. Furthermore, previous studies have shown that LPBN electrolytic lesions enhance baroreflex-mediated cardiovascular responses, which differs from the results of the present work [17,18]. The LPBN can relay autonomic information to other structures, which is crucial for the regulation of the autonomic function, enabling it to regulate the sympathetic and parasympathetic outflow to the cardiovascular system and promote changes in the heart rate and peripheral vascular resistance. The LPBN projects to the RVLM, and, according to Kubo et al., LPBN pressor site neurones mediate the cholinergic inputs responsible for the RVLM pressor responses [34]. Consequently, LPBN-reduced activity could act on silencing sympathetic pre-motor neurones by tackling the RVLM and, hence, indirectly, IML neurones, or by directly working on them.

Similar to our results, LPBN lesions in anaesthetised rats depress the peripheral chemoreflex response mainly by attenuating the shortening of the expiratory duration during hypoxia [35,36,37,38]. In addition, chemical or electrical stimulations within the LPBN and KF areas cause an increase in the blood pressure with changes in the breathing [15,18,35,36,37,38,39].

Located in the brainstem, the KF is a pontine nucleus that regulates respiration by modulating the respiratory rate and pattern, through its projections to the core respiratory nuclei in the brainstem, including the pre-Bötzinger complex, lateral parafacial nucleus, Bötzinger complex, and rostral ventral respiratory group projections [40]. Thus, the significant variation in the chemoreceptor function was not surprising because of the decreased sympathetic activity evoked by the KF modulation of the excitability and the absence of cardiovascular responses. The decrease in the neuronal excitability in the KF region showed that the affected neurones could be part of the central mechanisms involved in the chemosensory control of the sympathetic and respiratory chemoreflex. Moreover, the increase in the sympathetic nerve activity and phrenic nerve activity produced by the chemoreflex activation attenuated the chemical inhibition of KF neurones, suggesting that sympathoexcitation and increases in breathing are only partly mediated by the KF area [39,41]. The same effect was also observed after a blockade of the A5 neurons in the ventrolateral pons [42].

Similarly, other studies under anaesthesia have suggested that the KF area and ventrolateral pons are part of the central chemoreflex pathway [41,43,44]. The present study also tested the chemo- and baroreflex under anaesthesia. Therefore, the depressant effect of the agent cannot be neglected, as it may have exacerbated the reduced excitability of these neurones and altered the usual pattern of cardiovascular control.

A possible explanation for the sympathoexcitation reduction in the SHRs elicited by the LVV-hKir2.1 microinjection in the KF area is the reduced excitability of the presympathetic and respiratory neurons from the KF area to the ventrolateral medulla neurons involved in autonomic and respiratory control [41,42,45,46].

Regarding the parasympathetic system, and given that LPBN, PAG, and KF are all sympathetic nuclei, it was not surprising that an indirect assessment of the parasympathetic autonomic nervous system did not reveal any changes in the vagal tonus, which further highlights the restricted effect of LVV-hKir2.1 on the specific brain areas of interest.

Thus, in the present study, we showed the cardiovascular functional consequences of a persistently decreased excitability in the PAG, LPBN, and KF under hypertensive conditions. The observed responses suggested that the involvement of the PAG is putatively minor, whereas the LPBN has a clear input to the sympathoexcitation observed in hypertension. Our results also support the hypothesis that the LPBN and KF neurones activate facilitatory mechanisms that are critical to controlling the respiration and sympathetic outflow during chemoreceptor activation.

## 5. Limitations

Several limitations of this study should be considered when interpreting its results. Firstly, the study was conducted on a specific strain of rats, spontaneously hypertensive rats (SHRs), which may limit the generalizability of the findings to other strains or species. Although SHRs are a widely accepted model for studying hypertension, they may not fully represent the complexity of the disease in humans. Indeed, genome-wide association studies in human populations have successfully identified numerous genetic variations associated with an increased risk of hypertension and/or its complications. As a result, researchers now use knock-out/knock-in animal models to gain insight into the precise pathophysiological roles of specific genes in the development of these diseases [47].

Second, the study relied on a successful implantation of radio-telemetry probes and central microinjections into specific brain regions. These procedures require a high degree of precision and any slight deviations could potentially have affected the results. Although the researchers took measures to control the volume of the microinjection to limit the transduction to the confines of the chosen area, there is still the possibility of off-target effects.

Third, this study used lentiviral vectors for gene delivery, which, while efficient, can integrate into the host genome and potentially cause insertional mutagenesis. Although the researchers used a binary system to express the eGFP and human inwardly rectifying potassium channels of the hKir2.1 type in neuronal cells, the long-term safety of this approach remains a concern.

Fourth, the study’s metabolic evaluation was based on a 24 h period in metabolic cages. This short-term evaluation might not have fully captured the long-term metabolic changes in the animals.

Fifth, the study used a spectral analysis of the systolic blood pressure and RR interval data to assess the overall autonomic tone. Although this method is commonly used, it provides indirect measures of the sympathetic and parasympathetic activity and may not fully reflect the complex interplay of these systems in vivo.

Finally, the study evaluated the circadian patterns of the blood pressure and heart rate by segregating the telemetric data recordings into light and dark phases. However, this approach assumed that the activity patterns of the rats strictly followed the light–dark cycle, which may not always have been the case.

Despite these limitations, this study provided valuable insights into the roles of specific brain regions in the regulation of the cardiovascular function and offers promising avenues for future research. Future studies could benefit from using various animal models, refining surgical procedures, and employing more direct measures of autonomic activity. Moreover, it is interesting to note that the blood pressure and chronotropic changes at the KF level could be of interest for the mechanistic explanation of the importance of this nucleus within the circadian basis of cardiorespiratory- and sleep-related changes, especially regarding the non-rapid eye movement (NREM) sleep dynamics observed in individuals with hypertension [40,48]. This could help to define new therapeutic targets for hypertensive patients with sleep disorders such as insomnia, sleep apnoea, or comisa. It is also noteworthy that it is possible to return the blood pressure levels to normal or near-normal levels in elderly and hypertensive individuals. By studying functional changes over time, it was indeed possible in the study to show that older animals can improve their homeostatic state by genetically altering the excitability of the cells in the key areas of their brain responsible for homeostatic control. These results offer clues for future scientific research into reversing the deleterious functional effects that occur with age.

## 6. Conclusions

This study demonstrated that the targeted manipulation of the neuronal activity in specific brain regions through the use of lentiviral vectors can significantly impact the cardiovascular and respiratory functions in spontaneously hypertensive rats. These results suggested that the Lateral Parabrachial Nucleus (LPBN), Kolliker-Fuse Nucleus (KF), and Periaqueductal Grey Matter (PAG) play crucial roles in modulating these physiological processes.

These findings provided valuable insights into the neural mechanisms underlying hypertension and respiratory disorders, potentially paving the way for novel therapeutic strategies. However, further research is needed to fully understand the implications of these results and translate these findings into clinical applications.

## Figures and Tables

**Table 1:** Blood pressure (mmHg) and heart rate (bpm) circadian variation for PAG and control animals, before and 59 days after microinjection. Values are expressed as mean ± SEM. Abbreviations: sBP, systolic blood pressure; dBP, diastolic blood pressure; mBP, mean blood pressure; HR, heart rate; PAG, spontaneously hypertensive rats microinjected with LVV-hKir2.1 into the periaqueductal grey matter; and Sham, spontaneously hypertensive rats microinjected with LVV-eGFP into the rostral ventrolateral medulla.*, **, and *** correspond to significant (*p* < 0.05), very significant (*p* < 0.009), and extremely significant (*p* < 0.0005) difference between day 0 and day 60 PAG animals.

	Light Phase				Dark Phase			
Group	sBP	dBP	mBP	HR	sBP	dBP	mBP	HR
**Basal conditions**								
**PAG**	149 ± 2	126 ± 3	134 ± 3	309 ± 6	156 ± 3	133 ± 5	141 ± 4	343 ± 8
**SHAM**	157 ± 5	130 ± 5	139 ± 5	289 ± 7	160 ± 5	133 ± 6	142 ± 6	316 ± 8
**60 days after microinjection**								
**PAG**	164 ± 2 ***	140 ± 4 *	148 ± 3 **	284 ± 4 ***	172 ± 3 **	148 ± 7	156 ± 5 *	322 ± 5 **
**SHAM**	170 ± 12	141 ± 12	151 ± 11	268 ± 4	175 ± 11	147 ± 11	156 ± 11	311 ± 3

**Table 2:** Blood pressure (mmHg) and heart rate (bpm) circadian variation in KF and control animals before and 59 days after microinjection. Values are expressed as the mean ± SEM. Abbreviations: sBP, systolic blood pressure; dBP, diastolic blood pressure; mBP, mean blood pressure; HR, heart rate; KF, spontaneously hypertensive rats microinjected with LVV-hKir2.1 into the Kolliker-Fuse nucleus; and Sham, spontaneously hypertensive rats microinjected with LVV-eGFP into the rostral ventrolateral medulla. * Statistical significance for comparison between KF days 0 and 60.

	Light Phase				Dark Phase			
Group	sBP	dBP	mBP	HR	sBP	dBP	mBP	HR
**Basal conditions**								
**KF**	148 ± 2	127 ± 3	134 ± 3	322 ± 9	152 ± 5	131 ± 4	138 ± 4	353 ± 8
**SHAM**	157 ± 5	131 ± 6	140 ± 5	295 ± 5	160 ± 5	134 ± 6	143 ± 6	321 ± 6
**60 days after microinjection**								
**KF**	161 ± 6 *	138 ± 5 *	146 ± 5 *	294 ± 14 *	166 ± 7 *	142 ± 6 *	150 ± 6 *	327 ± 7 *
**SHAM**	177 ± 10	148 ± 12	158 ± 11	266 ± 5	181 ± 11	153 ± 13	163 ± 12	305 ± 8

## Data Availability

The datasets generated and analysed during the current study are not publicly available due to privacy and ethical considerations but are available from the corresponding author on reasonable request. All requests for access to the study data should be directed to the corresponding author, who will review requests on a case-by-case basis. Restrictions may apply, subject to the nature of the request, and the confidentiality and privacy rights of the study participants.

## References

[1] Esler M., Lambert E., Schlaich M. (2010). Point: Chronic activation of the sympathetic nervous system is the dominant contributor to systemic hypertension. J. Appl. Physiol..

[2] Grassi G., Ram V.S. (2016). Evidence for a critical role of the sympathetic nervous system in hypertension. J. Am. Soc. Hypertens..

[3] Rapp J.P. (1982). Dahl salt-susceptible and salt-resistant rats. A review. Hypertension.

[4] Dampney R.A. (1994). Functional organization of central pathways regulating the cardiovascular system. Physiol. Rev..

[5] Potts P.D., Paton J.F. (2006). Residual depressor function within RVLM of the ‘decentralized’ rat. J. Physiol..

[6] Geraldes V., Goncalves-Rosa N., Liu B., Paton J.F., Rocha I. (2014). Essential role of RVL medullary neuronal activity in the long term maintenance of hypertension in conscious SHR. Auton. Neurosci..

[7] Geraldes V., Gonçalves-Rosa N., Liu B., Paton J.F., Rocha I. (2014). Chronic depression of hypothalamic paraventricular neuronal activity produces sustained hypotension in hypertensive rats. Exp. Physiol..

[8] Kc P., Dick T.E. (2010). Modulation of cardiorespiratory function mediated by the parabrachial nucleus: Role of nitric oxide. Respir. Physiol. Neurobiol.

[9] Silvani A., Calandra-Buonaura G., Dampney R.A., Cortelli P. (2016). Brain-heart interactions: Physiology and clinical implications. Philos. Trans. R. Soc. A Math. Phys. Eng. Sci..

[10] Benarroch E.E. (2012). Periaqueductal gray: An interface for behavioral control. Neurology.

[11] Sato M., Shirasaka T., Uchida S., Shibamoto T. (2020). Neural Interaction between Respiratory and Autonomic Centers in the Medulla. Neurosci. Insights.

[12] Subramanian H.H. (2018). Distinct Periaqueductal Gray Projections to Medullary Cardioinhibitory and Excitatory Autonomic Pools in the Rat. Brain Res..

[13] Ciriello J., Caverson M.M., McMurray J.C. (2013). Medullary pathways mediating specific sympathetic responses to activation of dorsomedial hypothalamus. Neuroscience.

[14] Scopinho A.A., Antunes V.R., Mauad H., Reis D.G., Moraes D.J.A. (2016). The role of the parabrachial complex in the cardiorespiratory response evoked from hypothalamic defense area stimulation. Brain Res..

[15] Farnham M.M.J., Pilowsky P.M. (2018). The role of the lateral parabrachial nucleus in cardiovascular regulation. Auton. Neurosci. Basic Clin..

[16] Hayward L.F., Felder R.B. (1998). Lateral parabrachial nucleus modulates baroreflex regulation of sympathetic nerve activity. Am. J. Physiol. Regul. Integr. Comp. Physiol..

[17] Cisternas J.R., Lara J.P., Fuentealba J.A., Bonansco C., Díaz-Véliz G. (2020). Cardiovascular changes induced by microinjections of GABAergic agonists into the central nucleus of the amygdala and lateral parabrachial nucleus in the rat. Physiol. Rep..

[18] Silva-Carvalho L., Dawid-Milner M.S., Goldsmith G.E., Spyer K.M. (1991). The effects of electrical stimulation and lesions in the parabrachial area on arterial blood pressure and heart rate in anaesthetized cats. J. Auton. Nerv. Syst..

[19] Teschemacher A.G., Wang S., Lonergan T., Duale H., Waki H., Paton J.F.R., Kasparov S. (2005). Targeting specific neuronal populations using adeno-and lentiviral vectors: Applications for imaging and studies of cell function. Exp. Physiol..

[20] Duale H., Waki H., Howorth P., Kasparov S., Teschemacher A.G., Paton J.F. (2007). Restraining influence of A2 neurons in chronic control of arterial pressure in spontaneously hypertensive rats. Cardiovasc. Res..

[21] Liu B., Paton J.F., Kasparov S. (2008). Viral vectors based on bidirectional cell-specific mammalian promoters and transcriptional amplification strategy for use in vitro and in vivo. BMC Biotechnol..

[22] Paxinos G., Watson C. (2007). The Rat Brain in Stereotaxic Coordinates/George Paxinos.

[23] Gordon F.J., Matsuguchi H., Mark A.L. (1981). Abnormal baroreflex control of heart rate in prehypertensive and hypertensive Dahl genetically salt-sensitive rats. Hypertension.

[24] Silva Carvalho L., Moniz De Bettencourt J., Esguelha N., Marques M., Silva Carvalho J. (1987). The Double Reflexogenic Action of Lobeline, Acetylcholine and Cyanides on the Carotid Body, Influence of Phentolamine and Sulpiride. Chemoreceptors in Respiratory Control.

[25] Tavares C., Carneiro R., Laranjo S., Rocha I. Computational tools for assessing cardiovascular variability. Proceedings of the 1st Portuguese Meeting in Bioengineering.

[26] Castro R., Ramalho S., Nóbrega A. (2000). Selection of RR interval in the electrocardiogram to determine the respiratory sinus arrhythmia. Rev. Bras. Med. Esporte.

[27] Dampney R.A. (2016). Central Neural Control of the Cardiovascular System: Current Perspectives. Adv. Physiol. Educ..

[28] Mortensen L.H., Ohman L.E., Haywood J.R. (1994). Effects of lateral parabrachial nucleus lesions in chronic renal hypertensive rats. Hypertension.

[29] Lovick T.A. (1993). The periaqueductal gray-rostral medulla connection in the defence reaction: Efferent pathways and descending control mechanisms. Behav. Brain Res..

[30] Carrive P. (1993). The periaqueductal gray and defensive behavior: Functional representation and neuronal organization. Behav. Brain Res..

[31] Schenberg L.C., Lucas Brandão C.A., Vasquez E.C. (1995). Role of periaqueductal gray matter in hypertension in spontaneously hypertensive rats. Hypert.

[32] Guyenet P.G. (2006). The sympathetic control of blood pressure. Nat. Rev. Neurosci..

[33] Jun S., Ou X., Shi L., Yu H., Deng T., Chen J., Nie X., Hao Y., Shi Y., Liu W. (2023). Circuit-Specific Control of Blood Pressure by PNMT-Expressing Nucleus Tractus Solitarii Neurons. Neurosci. Bull..

[34] Kubo T., Fukumori R., Kobayashi M., Yamaguchi H. (1998). Evidence suggesting that lateral parabrachial nucleus is responsible for enhanced medullary cholinergic activity in hypertension. Hypertens. Res..

[35] Song G., Poon C.S. (2009). Lateral parabrachial nucleus mediates shortening of expiration during hypoxia. Respir. Physiol. Neurobiol..

[36] Chamberlin N.L., Saper C.B. (1994). Topographic organization of respiratory responses to glutamate microstimulation of the parabrachial nucleus in the rat. J. Neurosci..

[37] Molkov Y.I., Bacak B.J., Dick T.E., Rybak I.A. (2017). Control of breathing by interacting pontine and pulmonary feedback loops. Front. Neural Circ..

[38] Morris K.F., Nuding S.C., Segers L.S., Baekey D.M., Shannon R., Lindsey B.G., Dick T.E. (2010). Respiratory and Mayer wave-related discharge patterns of raphe and pontine neurons change with vagotomy. J. Appl. Physiol..

[39] Damasceno R.S., Takakura A.C., Moreira T.S. (2014). Regulation of the chemosensory control of breathing by Kölliker-Fuse neurons. Am. J. Physiol.-Regul. Integr. Comp. Physiol..

[40] Varga A.G., Whitaker-Fornek J.R., Maletz S.N., Levitt E.S. (2022). Activation of orexin-2 receptors in the Kӧlliker-Fuse nucleus of anesthetized mice leads to transient slowing of respiratory rate. Front. Physiol..

[41] Damasceno R.S., Takakura A.C., Moreira T.S. (2015). Respiratory and sympathetic chemoreflex regulation by Kölliker-Fuse neurons in rats. Pflüg. Arch. Eur. J. Physiol..

[42] González-García M., Carrillo-Franco L., Peinado-Aragonés C.A., Díaz-Casares A., Gago B., López-González M.V., Dawid-Milner M.S. (2023). Impact of the glutamatergic neurotransmission within the A5 region on the cardiorespiratory response evoked from the midbrain dlPAG. Pflüg. Arch. Eur. J. Physiol..

[43] Koshiya N., Guyenet P.G. (1994). Role of the pons in the carotid sympathetic chemoreflex. Am. J. Physiol.-Regul. Integr. Comp. Physiol..

[44] Rosin D.L., Chang D.A., Guyenet P.G. (2006). Afferent and efferent connections of the rat retrotrapezoid nucleus. J. Comp. Neurol..

[45] Silva J.N., Lucena E.V., Silva T.M., Damasceno R.S., Takakura A.C., Moreira T.S. (2016). Inhibition of the pontine Kölliker-Fuse nucleus reduces genioglossal activity elicited by stimulation of the retrotrapezoid chemoreceptor neurons. Neuroscience.

[46] Guyenet P.G., Stornetta R.L. (2022). Rostral ventrolateral medulla, retropontine region and autonomic regulations. Auton. Neurosci..

[47] Ohara H., Nabika T. (2022). Genetic Modifications to Alter Blood Pressure Level. Biomedicines.

[48] Bonis J.M., Neumueller S.E., Krause K.L., Kiner T., Smith A., Marshall B.D., Qian B., Pan L.G., Forster H.V. (2010). A role for the Kölliker-Fuse nucleus in cholinergic modulation of breathing at night during wakefulness and NREM sleep. J. Appl. Physiol..

